# Preparing Ambient-Processed Perovskite Solar Cells with Better Electronic Properties via Preheating Assisted One-Step Deposition Method

**DOI:** 10.1186/s11671-020-03407-9

**Published:** 2020-09-16

**Authors:** Xi Zhang, Wenyao Yang, Jingjing Qi, Yinggang Hu

**Affiliations:** 1 School of Electronics and Information Engineering, Sichuan Technology and Business University, Chengdu, 611745 Sichuan China; 2 Chongqing Engineering Research Center of New Energy Storage Devices and Application, Chongqing, 402160 People’s Republic of China; 3 State Key Laboratory of Electronic Thin Films and Integrated Devices, University of Electronic Science and Technology of China (UESTC), Chengdu, 610054 Sichuan China; 4 School of Optoelectronic Science and Engineering, University of Electronic Science and Technology of China (UESTC), Chengdu, 610054 Sichuan China

**Keywords:** Perovskite solar cells, Ambient condition, Crystallinity, Heat-assisted one-step method

## Abstract

Although the power conversion efficiency (PCE) of perovskite solar cells (PSCs) increases rapidly, there are still some issues that limit their commercialization. The perovskite is sensitive to the water molecules, increasing the difficulty in the preparation of perovskite films in ambient condition. Most high-performance PSCs based on conventional method are required to be prepared in inert atmosphere condition, which increase the fabrication cost. To fabricate the high-quality perovskite in ambient condition, we preheated the substrates and selected the proper anti-solvent. As a result, the target perovskite films show a better crystallinity compared with perovskite film prepared via the conventional one-step deposition method in ambient condition. The PSCs prepared in ambient condition yield the improved PCE of 16.89% from a PCE of 11.59%. Compared with the reference devices, the performance stability of target PSCs is much better than that of reference PSCs.

## Introduction

Perovskite solar cells (PSCs) have been attracting a lot of attention since the organic-inorganic hybrid perovskite was used as the light harvester of solar cells [1–5]. The perovskite film shows numerous excellent photoelectrical properties such as high light absorption coefficient, suitable bandgap, and good charge transport. The latest reported certified highest power conversion efficiency (PCE) reached 25.2% (https://www.nrel.gov/pv/assets/pdfs/best-research-cell-efficiencies.20200311.pdf).

The conventional structure of PSCs contains charge transport layers, light harvester layer, and electrodes [6–11]. The light harvester layer inserted between charge transport layers is vital for photovoltaic performance of PSCs. Perovskite films are composed of numerous submicron-sized crystal grains, and the size of these grains is related to the preparation process of perovskite films. Most of the perovskite films were prepared in dry air or inert gas condition to avoid the affection of water molecules on the crystallization process of perovskite films. However, preparing perovskite films in inert gas condition or dry air would increase the fabrication cost, which is unfavored for the commercialization of PSCs. Since 2014, some research groups started to study the preparation method of PSCs in ambient condition [12–23]. They fabricated the PSCs using two-step method, and they optimized the ingredient and deposition process of perovskite films. The highest PCE reached 16%. Miyasaka group fabricated the mesoporous type PSCs based on CH_3_NH_3_PbI_3_ under the condition with a relative humidity of 30% at 25 °C [22]. The devices yielded a PCE of 15.3% and had a good reproducibility. The devices stored in dry-air and dark condition for 1 month retained 80% of the initial PCE. In 2015, Mori et al., from Aichi Institute of Technology, used gas flow assist method to deposit the perovskite films in air condition (relative humidity of 42–48% at 25 °C) [24]. The planar type PSCs based on these perovskite films has a PCE of 16.32% and 13.31% under reverse and forward scanning direction, respectively. Ko et al. fabricated mesoporous type CH_3_NH_3_PbI_3_ based PSCs in the condition with different humidity, and the devices prepared under the condition with a relative humidity of 50% at 23.1 °C show the best photovoltaic performance with the highest PCE of 15.76% [14]. They used the tow-step deposition method, and the substrates were preheated before the spin-coating of PbI_2_ solution whose solvent was dimethylformamide (DMF). The obtained PbI_2_ had an enhanced transparency, and the PCE of devices has been increased to 15% from 10%. Enhanced preheat temperature induced the increase in grain sizes of perovskite films, but the residual PbI_2_ became more. Hence, it is important to find the proper preheat temperature to balance the crystal size and transfer efficiency of PbI_2_. In 2017, Cheng et al. increased the vapor pressure of the solvent to reduce the ingress of oxygen and water molecules during the PbI_2_ deposition through preheating the substrate before deposition of PbI_2_ films [12]. They obtained an air-processed PSCs fabricated under a humidity of 70% RH, and the PCE reached 18.11%. There are some reports that the water molecules can improve the crystallization quality of perovskite films during the annealing step of the perovskite films when the one-step method is used to prepare the perovskite films. In 2014, You et al. found that the PCE of planar type PSCs based on CH_3_NH_3_PbI_3-X_Cl_x_ improved under the particular humidity [23]. The perovskite films were annealed for 1 h under the relative humidity of 30 ± 5% at room temperature, which increase the PCE to 16.6%. The result also clarified that the proper humidity was beneficial to the formation of the more compact perovskite. A lot of study demonstrated that the relative humidity should be lower than 80% during the preparation of the perovskite. In 2015, Lv et al. from Changzhou University used dimethylacetamide as the solvent of the perovskite [15]. This solvent can accelerate the crystallization of the CH_3_NH_3_PbI_3_ perovskite so that the affection of humidity on perovskite films would be decreased dramatically. Therefore, devices with the champion PCE of 16.15% were obtained under the condition with a relative humidity of 28% at room temperature. In 2016, Sveinbjornsson et al. also preheated the substrate and optimized the temperature at range between 20 and 100 °C in ambient condition [19]. The PSCs based on (FAPbI_3_)_1-x_(MAPbBr_3_)_x_ with a preheat temperature of 50 °C yielded an average PCE of 17.6%. In 2019, Li et al. optimized the preheat temperature and anti-solvent dropping time to fabricate the CH_3_NH_3_PbI_3_-based PSCs under the condition with a relative humidity of 90% at room temperature [25]. They obtained the devices with a PCE output of 19.5%.

Engineering the anti-solvent is another effective way to improve the photovoltaic performance of PSCs prepared in ambient condition. To avoid the affection of the moisture on the perovskite formation, the anti-solvent selection is very important. The commonly used anti-solvent includes chlorobenzene, diethyl ether, and ethyl acetate. Troughton et al. thought the ethyl acetate acted as both anti-solvent and moisture absorber material which reduce the affection of water molecules, so the ethyl acetate solvent is superior compared with other anti-solvent such as chlorobenzene and diethyl ether.

Here, we used preheating method in one-step deposition process when preparing perovskite films in ambient condition (relative humidity of 25–30% at 20 °C). We also used ethyl acetate solvent as the anti-solvent as the substitution to diethyl ether. The preheated substrate can accelerate the evaporation of the solvent, which can reduce the ingress of oxygen and moisture. Furthermore, diethyl ether can not only extract the solvent of perovskite but also absorb the water molecules. The target PSCs yield a better PCE of 16.89% compared with the reference PSCs. Compared with other fabrication methods, this method is more cost-effective and simpler. It does not need a complicated process.

## Methods

### Materials

All of the materials were purchased form Ying Kou You Xuan Trade Co. Ltd, if not specified. DMF and dimethyl sulfoxide (DMSO) were purchased from Sigma-Aldrich Corp. The SnO_2_ nanoparticle colloidal solution was purchased from Alfa Aesar. The CH_3_NH_3_PbI_3_ solution was prepared by mixing PbI_2_, CH_3_NH_3_I, and DMSO into DMF according to ref. [26]. The HTL solution was prepared by dissolving 72.3 mg (2,29,7,79-tetrakis(N,N-di-p-methoxyphenylamine)-9,9-spirobifluorene) (spiro-MeOTAD), 28.8 μL 4-tert-butylpyridine, 17.5 μL of a stock solution of 520 mg/mL lithium bis(trifluoromethylsulfonyl)imide in acetonitrile, and 29 μL of a solution of 300 mg/ml FK209 in acetonitrile in 1 ml chlorobenzene.

### Preparation

**Fig. 1:**
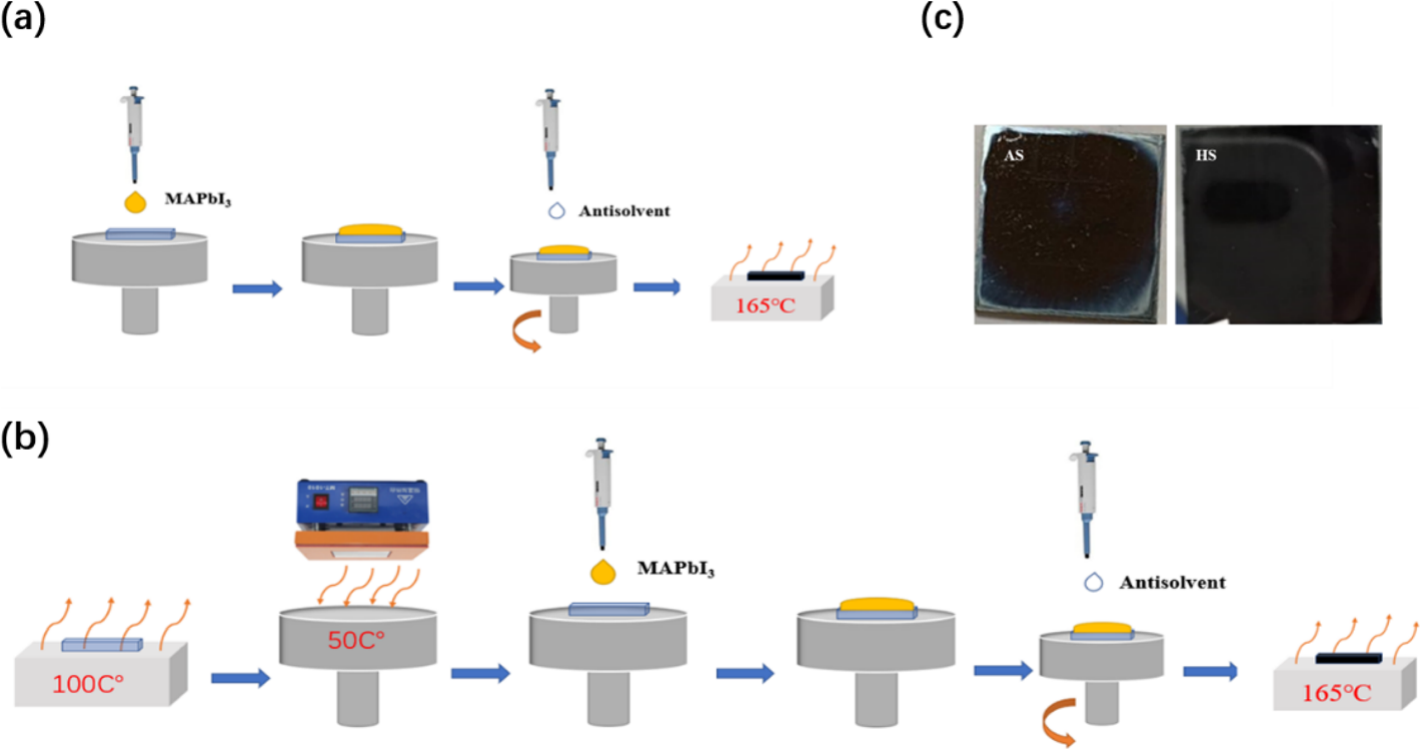
**a** Anti-solvent spin-coating method of perovskite film, **b** heat anti-solvent spin-coating method of perovskite film, **c** photograph of perovskite deposited with different methods

The ITO glasses were cleaned sequentially in acetone, absolute ethyl alcohol, and deionized water ultrasonic bath for 15 min, respectively. After ITO glasses were cleaned by the UV-Ozone treat for 20 min, a SnO_2_ film was deposited by spin-coating diluted SnO_2_ nanoparticle colloidal solution (Alfa Aesar (tin(IV) oxide, 15% in H_2_O colloidal dispersion)) according to ref. [27]. After the spin-coating, the SnO_2_ film was heated at 165 °C for 0.5 h. Then, the substrates were treated with the UV-Ozone again and transferred into the glovebox. Perovskite films were prepared according to Fig. 1. The HTL was prepared by spin-coating the HTL solution at 5000 rpm for 30 s. Finally, 100 nm of Au top electrode was thermally evaporated onto the HTL.

### Characterization

The current density-voltage (J-V) curves of PSCs was recorded by Keithley source unit 2400 under AM 1.54G sun intensity illumination by a solar silmulator from Newport Corp. The X-ray diffraction patterns were recorded with Bruker D8 ADVANCE A25X. Scanning electron microscope (SEM) was conducted on field emission fitting SEM (FE-Inspect F50, Holland). The absorption of perovskite was measured using Shimadzu 1500 spectrophotometer. Statistical data is plotted using box chart.

## Result and Discussion

The process of the conventional anti-solvent spin-coating method (AS) and heat antisolvent spin-coating method (HS) is shown in Fig. 1 a and b, respectively. Compared with AS, the substrates and mount for spin-coating need be preheated before the perovskite solution is dropped onto substrates. The anti-solvent is dropped onto the sample surface during the spin-coating process. After the spin-coating, the samples are transferred onto a heating plate with a temperature of 165 °C. The dropping of the anti-solvent is finished before the film become turbid. The photographs of perovskite films prepared with different methods are shown in Fig. 1c. Here, the diethyl ether and ethyl acetate are used as the anti-solvent. Compared with diethyl ether, the ethyl acetate is more suitable for perovskite deposition in ambient condition. Ethyl acetate can absorb the water molecules and protect the perovskite film from water penetrating. Here, the perovskite films prepared with AS and HS method are referred to AS-perovskite and HS-perovskite, respectively.

**Fig. 2:**
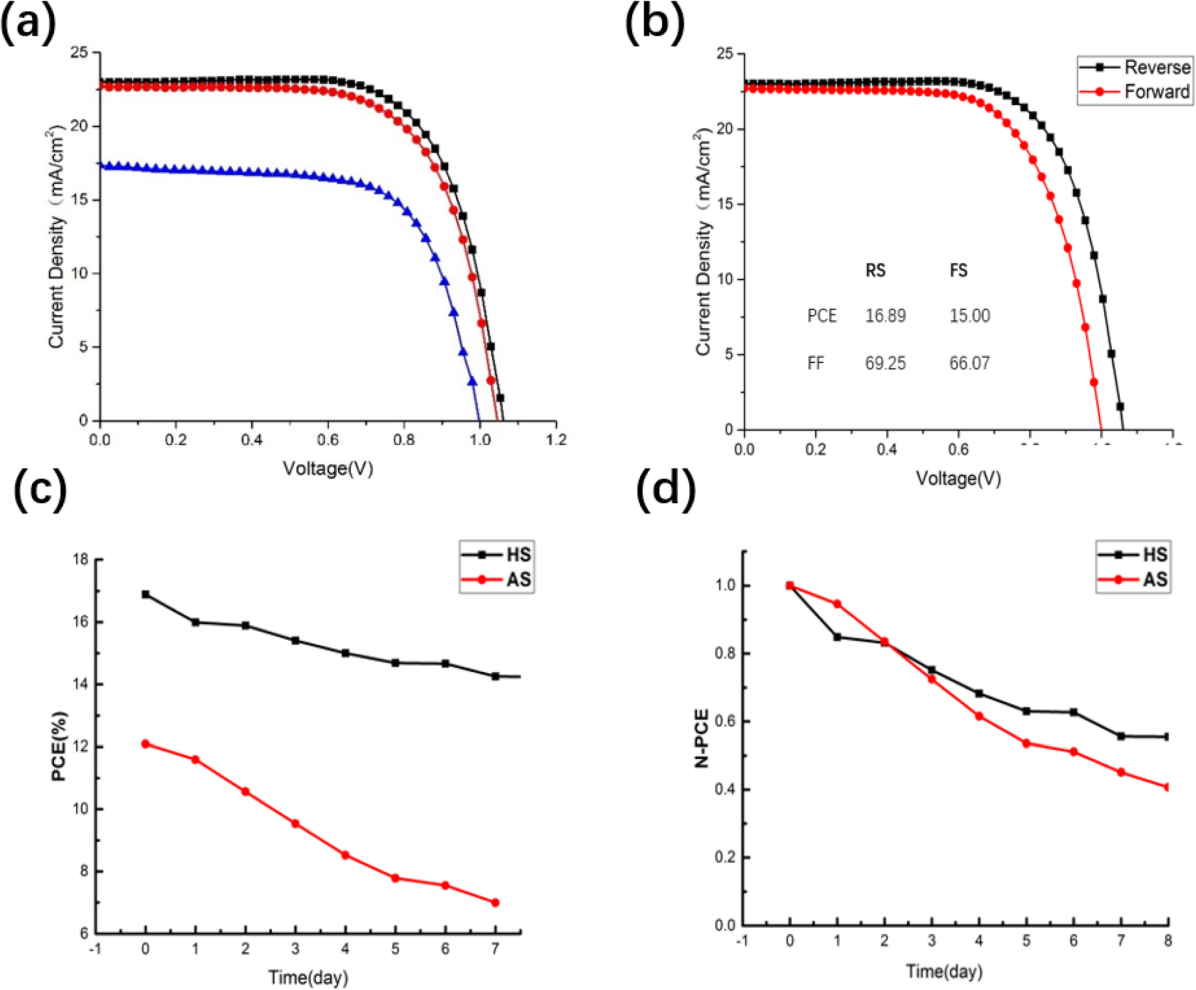
**a** J-V cures of PSCs based on different perovskite (black line: HS EA, red line: HS DE, blue line: AS EA) (HS EA stands for PSCs based on perovskite prepared via preheating method with an anti-solvent of ethyl acetate, HS DE stands for PSCs based on perovskite prepared via preheating method with an anti-solvent of diethyl ether, AS EA stands for PSCs based on perovskite prepared via conventional method with an anti-solvent of ethyl acetate), **b** J-V curves of PSCs based on HS EA under different scanning direction, **c** PCE variation with time, and **d** normalized PCE variation with time

**Fig. 3:**
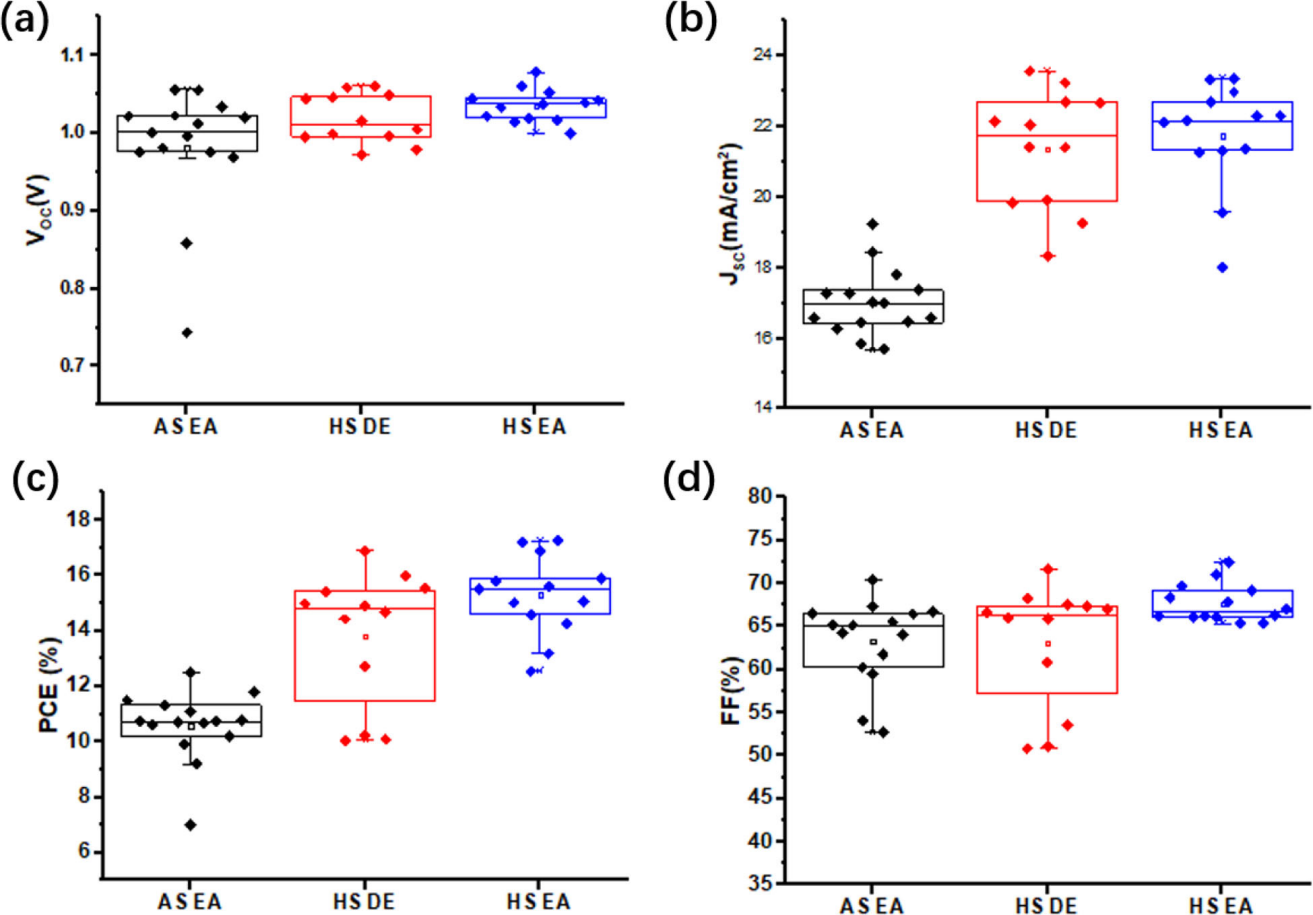
Statistic data of photovoltaic parameters including V_OC_ (**a**), J_SC_ (**b**), FF (**c**), and PCE (**d**)

Here, we fabricated PSCs based on HS-perovskite and AS-perovskite. The PSCs based on AS-perovskite (AS-PSCs) were used as the reference devices. There were two different anti-solvents including diethyl ether and ethyl acetate used in preparation process of HS-perovskite. Only ethyl acetate was used as the anti-solvent in preparation process of AS-perovskite. The current density versus voltage (J-V) curves for the best-performance devices in each group are shown in Fig. 2a, and the photovoltaic parameters are listed in Table 1. The statistical data of photovoltaic parameters for more than 15 devices in each group is shown in Fig. 3. The PSCs based on HS-perovskite (HS-PSCs) yield a much better photovoltaic performance compared with AS-PSCs. The PSCs based on perovskite films prepared with HS method and ethyl acetate (HS-EA-PSCs) have the highest power conversion efficiency (PCE) of 16.89% with an open-circuit voltage (V_OC_) of 1.06 V, short-circuit current density (J_SC_) of 22.98 mA/cm^2^, and fill factor (FF) of 69.25%. The hysteresis of the champion HS-EA-PSCs is shown in Fig. 2b. The PSCs based on perovskite films prepared with HS method and diethyl ether (HS-DE-PSCs) yield a PCE of 15.99%. The PCE for reference PSCs is 11.59% which is much lower than PCEs of HS-PSCs. From the J-V curves and statistical data, the main reason for the photovoltaic performance improvement in HS-PSCs is the obviously increased current density. To explore the mechanism for the photovoltaic performance improvement, several characterizations have been carried out on the perovskite films.

**Table 1:** Photovoltaic parameters of PSCs based on different perovskite

Devices	V_OC_(V)	J_SC_(mA/cm^2^)	FF (%)	PCE (%)
HS-EA-PSCs	1.06	22.98	69.25	16.89
HS-DE-PSCs	1.04	22.69	67.35	15.99
AS-PSCs	0.99	17.30	67.14	11.59

The stability of PSCs based on different perovskite films is also been characterized. The devices were stored under air condition, and the photovoltaic performance was measured every day. The PCE change with the time is shown in Fig. 2b. After 1 week, the PCE of HS-PSCs decreased to 14.25% from the initial PCE of 16.89%, and the value retained 84.3% of the initial PCE. However, the PCE of AS-PSCs dropped to 6.99% from 12.09%, and the value remained only 57.8% of the initial PCE value. The normalized PCE changes of different devices are shown in Fig. 2c. The stability results clarify that the HS-PSCs have a much better performance stability. The reason for the better stability will be discussed in following parts.

**Fig. 4:**
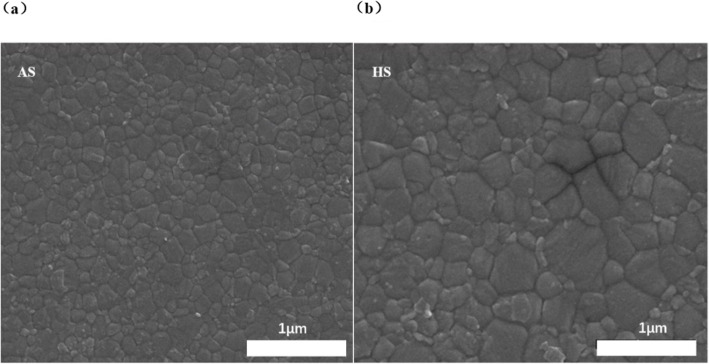
SEM images of perovskite prepared with AS method (**a**) and HS method (**b**)

**Fig. 5:**
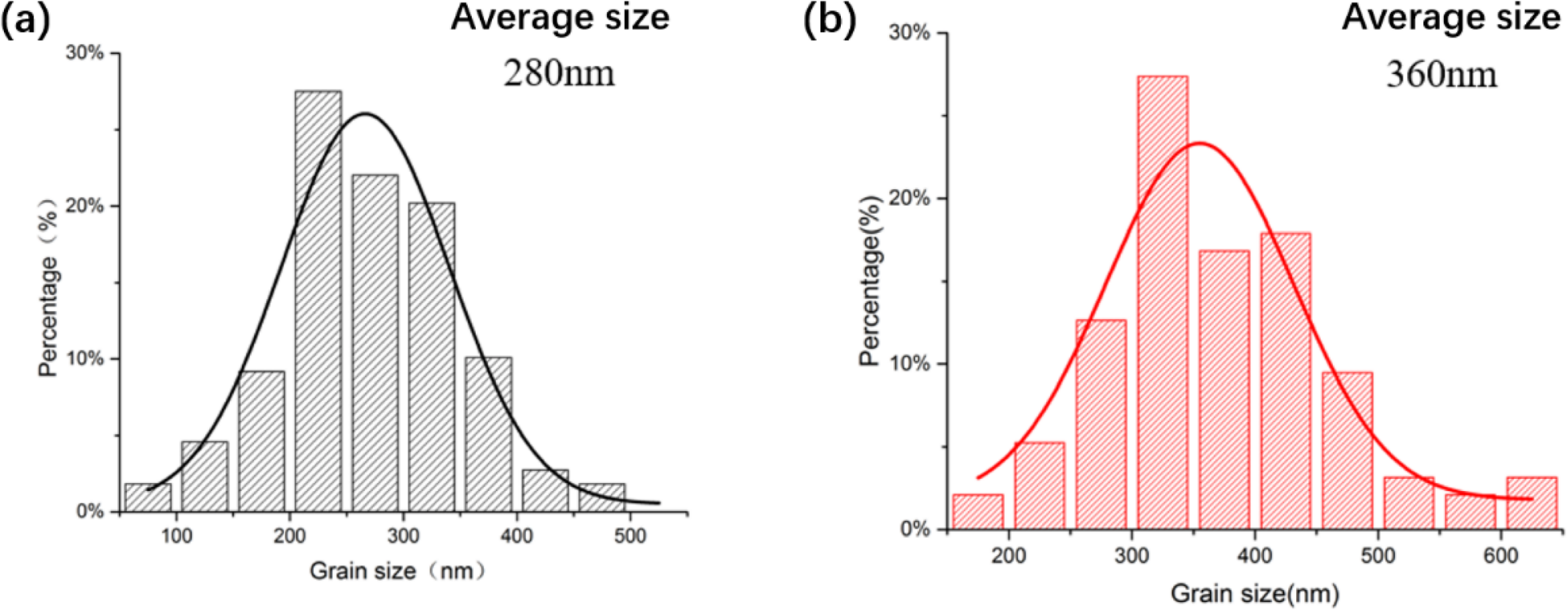
Grain size distribution of perovskite films prepared with AS method (**a**) and HS method (**b**)

The crystallinity and topography of the perovskite affect the photovoltaic performance of the PSCs. A compact and uniform perovskite film is essential for the excellent device performance. The compact light absorption layer can avoid the direct contact between electron transport layer and hole transport layer (HTL), and the uniform surface is beneficial to the complete coverage of the HTL, reducing the short-circuit loops inside devices. The scanning electron microscope (SEM) images of perovskite prepared with different methods are shown in Fig. 4. From the SEM images, the perovskite films are compact and uniform, and the crystal boundaries are clear. The perovskite film prepared with HS method shows a much larger average grain size inducing a less boundary and lower defect density. The distributions of the perovskite crystal size are shown in Fig. 5. The average size of the perovskite prepared with AS method and HS method is 280 nm and 360 nm, respectively. From Fig. 3, the proportion of crystal grains with a size more than 400 nm in HS-perovskite is much larger than that in AS-perovskite, which is consistent with the surface SEM image result. The larger crystal size results in a better moisture stability of perovskite films.

**Fig. 6:**
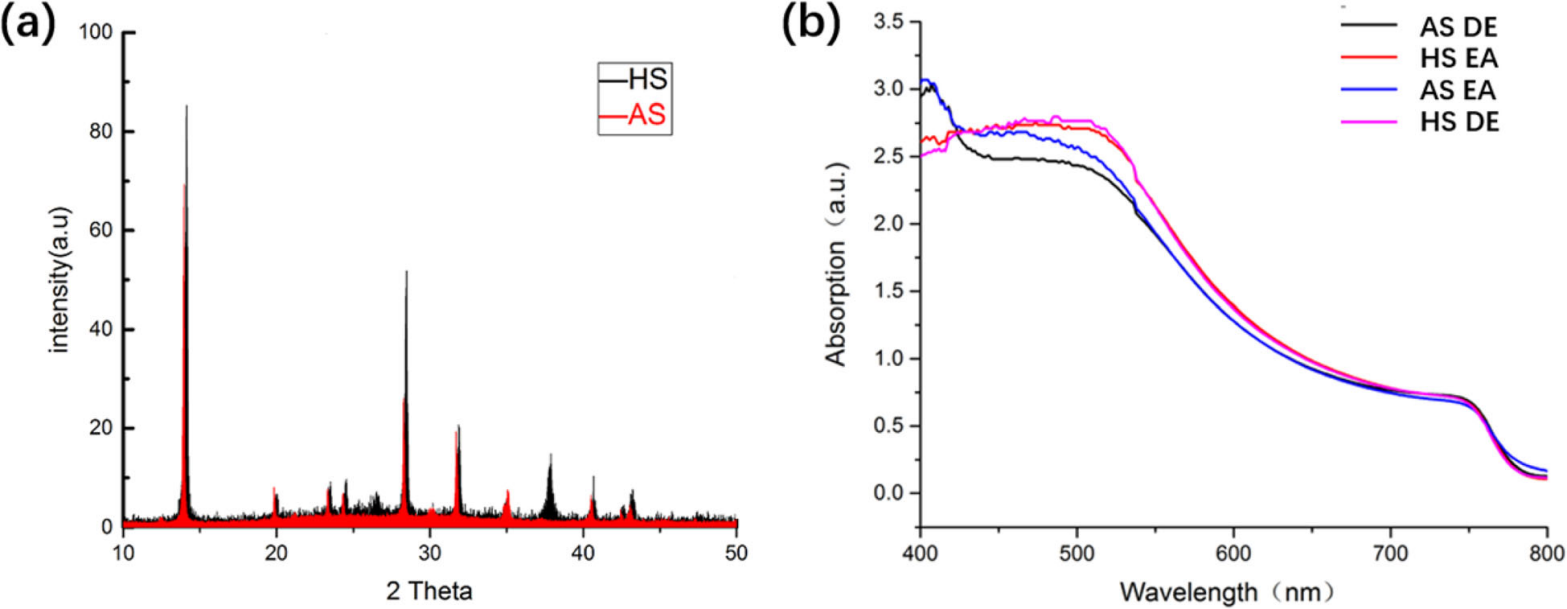
**a** XRD pattern of HS-perovskite and AS-perovskite. **b** UV-visible light absorption curves of different perovskite films

The crystallinity of perovskite films is characterized using x-ray diffraction (XRD) measurements. The XRD patterns are shown in Fig. 6. The peak located at 14.1°, 28.4° and 31.3° corresponds to (110), (220), and (310) plane of perovskite films, respectively. There are no apparent peaks around 12° in the XRD pattern, indicating that there is almost no PbI_2_ residue in both perovskite films. The perovskite film based on AS method with the anti-solvent of EA has a higher XRD peak, clarifying a better crystallinity.

The UV-visible light absorption measurement is conducted to characterize the light absorption capacity of perovskite prepared by different methods. The perovskite films apparent absorption when the incident light wavelength is below 770 nm. The absorption edges of perovskite films prepared with different methods overlap, demonstrating all perovskite films have a similar bandgap and the ingredient of perovskite films is not affected by the preparing methods. The absorption of HS-perovskite films is higher than that of AS-perovskite films in the wavelength range of 450–700 nm. The higher absorption of HS-perovskite films results in higher photo-induced-carrier density, leading to a higher current density in devices operated under the sunlight illumination.

## Conclusion

In summary, we used preheating assisted one-step method to fabricate high-quality perovskite films in ambient condition. We also compared the different anti-solvents to prepare the perovskite films. The target PSCs based on perovskite prepared HS method with an anti-solvent of EA showed the best photovoltaic performance with an improved PCE of 16.89% compared with that of reference PSCs. The enhanced photovoltaic performance results from the better crystallinity of HS-EA perovskite films. The better crystallinity of perovskite also results in a higher performance stability. This work has clarified that preheating assisted one-step method is an effective way to prepare perovskite films in ambient condition.

## Data Availability

All the data are fully available without restrictions.

## Abbreviations

PSCs: Perovskite solar cells
PCE: Power conversion efficiency
Spiro-MeOTAD: (2,29,7,79-tetrakis(N,N-di-p-methoxyphenylamine)-9,9-spirobifluorene)
DMSO: Dimethyl sulfoxide
DMF: Dimethylformamide
J-V: Current density-voltage
SnO_2_: Tin dioxide
SEM: Scanning electron microscope
ITO: Indium tin oxide
MA: CH_3_NH_3_
FA: HC(NH_2_)_2_
V_OC_: Open-circuit voltage
J_SC_: Short-circuit current density
FF: Fill factor
XRD: X-ray diffraction
HTL: Hole transport layer
AS: Conventional anti-solvent spin-coating method
HS: Heat anti-solvent spin-coating method
FK209: Tris(2-(1H-pyrazol-1-yl)-4-tert-butylpyridine)-cobalt(III) bis(trifluoromethylsulphonyl)imide
